# The impact of the senescent microenvironment on tumorigenesis: Insights for cancer therapy

**DOI:** 10.1111/acel.14182

**Published:** 2024-04-22

**Authors:** Wenqiang Zhang, Kexin Zhang, Junfeng Shi, Hongyan Qiu, Chengxia Kan, Yujie Ma, Ningning Hou, Fang Han, Xiaodong Sun

**Affiliations:** ^1^ Department of Endocrinology and Metabolism, Clinical Research Center, Shandong Provincial Key Medical and Health Discipline of Endocrinology Affiliated Hospital of Shandong Second Medical University Weifang China; ^2^ Department of Pathology Affiliated Hospital of Shandong Second Medical University Weifang China

**Keywords:** cancer, senescence‐associated secretory phenotype, senescent, tumor microenvironment

## Abstract

The growing global burden of cancer, especially among people aged 60 years and over, has become a key public health issue. This trend suggests the need for a deeper understanding of the various cancer types in order to develop universally effective treatments. A prospective area of research involves elucidating the interplay between the senescent microenvironment and tumor genesis. Currently, most oncology research focuses on adulthood and tends to ignore the potential role of senescent individuals on tumor progression. Senescent cells produce a senescence‐associated secretory phenotype (SASP) that has a dual role in the tumor microenvironment (TME). While SASP components can remodel the TME and thus hinder tumor cell proliferation, they can also promote tumorigenesis and progression via pro‐inflammatory and pro‐proliferative mechanisms. To address this gap, our review seeks to investigate the influence of senescent microenvironment changes on tumor development and their potential implications for cancer therapies.

## Graphical abstract

The senescence microenvironment promotes SASP secretion, which includes interleukins, chemokines, growth factors, inflammatory molecules, regulators, and proteases. These factors have the ability to exert bidirectional effects on tumor promotion or inhibition.

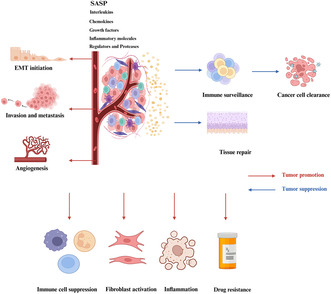

**Abbreviations**
CAFs: cancer‐associated fibroblasts
DDR: DNA damage response
DTCs: disseminated tumor cells
ECM: extracellular matrix
HA: hyaluronic acid
MSCs: mesenchymal stem cells
PDL1: programmed death‐ligand 1
RT: radiation therapy
ROS: reactive oxygen species
SASP: senescence‐associated secretory phenotype
TME: tumor microenvironment
TIS: therapy‐induced senescence

## 1 INTRODUCTION

**FIGURE 1:**
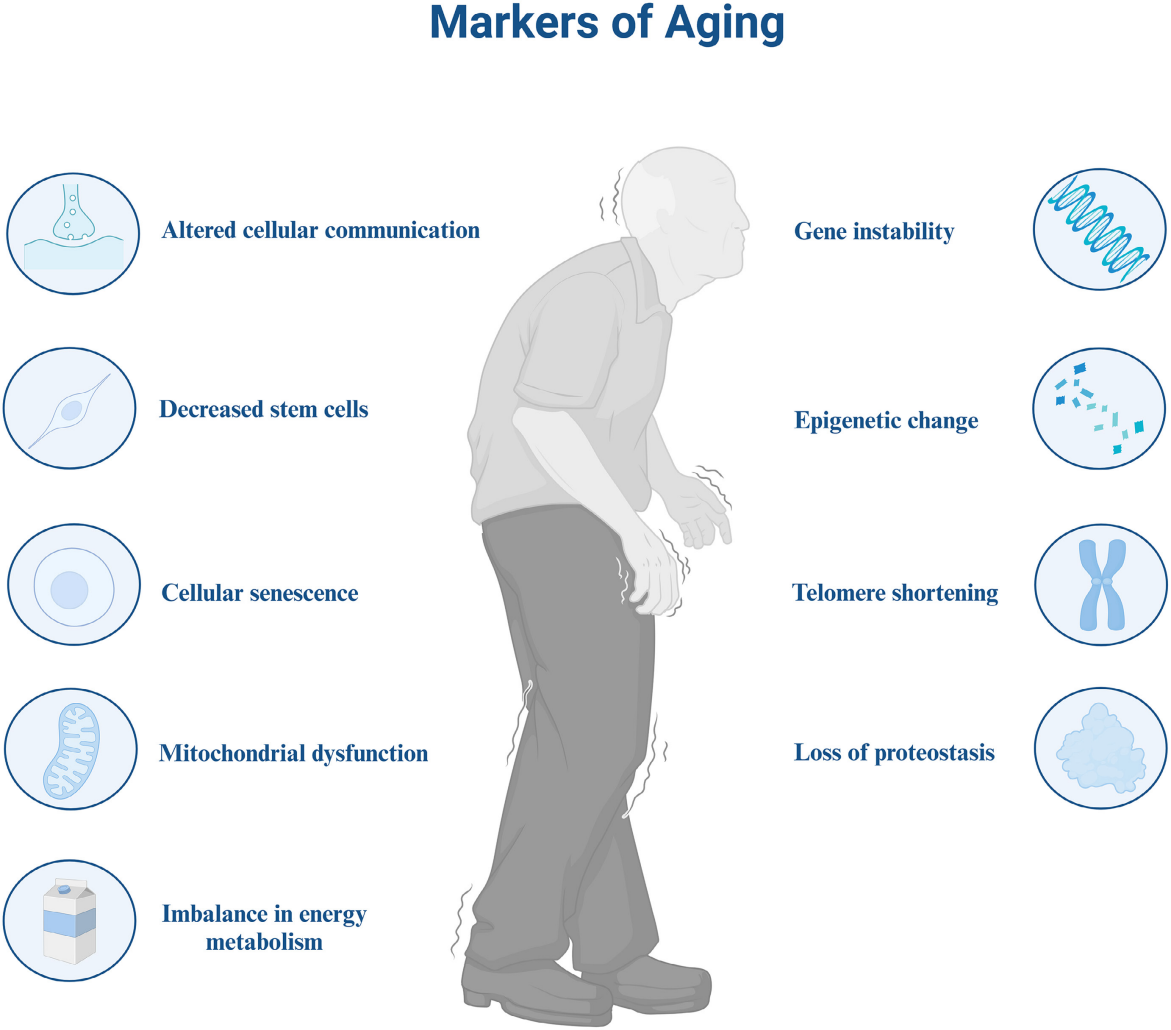
Signs of human aging. The nine hallmarks of human aging include altered cellular communication, stem cell exhaustion, cellular senescence, mitochondrial dysregulation, disturbed energy metabolism, genetic instability, epigenetic changes, telomere shortening, and loss of protein homeostasis.

Aging is an intricate and persistent process characterized by numerous factors and mechanisms that collectively lead to the gradual accumulation of senescent cells within the aging organism. This accumulation contributes to various dysfunctions and significantly increases the risk of developing age‐related diseases (Rossiello et al., 2022; Selman & Pardo, 2021). It has long been believed that there is a close connection between the aging process and the occurrence of tumors, particularly as cancer incidence tends to rise among the elderly population (Rozhok & DeGregori, 2019). However, upon examining the cellular level, we observe contrasting behaviors between senescent cells and tumor cells. Senescent cells, which are a hallmark of aging, exhibit a state of proliferative arrest and are more prone to undergo apoptosis (Figure 1). In contrast, tumor cells display the exact opposite behavior by evading cell cycle checkpoints and resisting programmed cell death (Zhao et al., 2023). This apparent contradiction compels us to explore the nuanced interplay between aging and tumorigenesis on a deeper level.

Historically, in 1961, Hayflick (1965) and Hayflick and Moorhead (1961) made a groundbreaking discovery while studying human diploid cell lines in continuous culture. They observed that fibroblasts, undergoing a limited number of cell divisions, reached an irreversible stagnation in their ability to multiply, a phenomenon now recognized as senescence. Aging imparts substantial changes to the microenvironment, which, in the context of tumor development, can lead to a profound remodeling of the tumor immune microenvironment. This remodeling occurs partly through the activation of the senescence‐associated secretory phenotype (SASP), a complex mixture of various cytokines and signaling molecules (Cuollo et al., 2020). The SASP can inadvertently contribute to tumor cell survival and provide an avenue for immune escape, thus fostering an environment conducive to tumorigenesis (Gajewski et al., 2013). The impact of aging extends beyond senescent cells, also uniquely affecting normal fibroblasts and immune cells within the tumor microenvironment (TME). Several changes resulting from aging likewise increase tumor aggressiveness and promote metastasis, thereby exacerbating the challenges of managing cancer in the elderly (Fane & Weeraratna, 2020).

Previous research has established that cancer and aging share common features, including genomic instability, telomere attrition, epigenetic alterations, impaired nutrient sensing, and mitochondrial dysfunction (Aunan et al., 2017). These commonalities are largely attributable to mutations in genes, driving both tumor proliferation and the aging process in organisms (Risques & Kennedy, 2018). Additionally, these mutations affect genes in growth pathways (Aunan et al., 2017). Rozhok and DeGregori (2019) advanced our understanding by integrating the contemporary multistage carcinogenesis model with differential senescence‐dependent somatic cell selection, offering a more comprehensive explanation for the escalating cancer incidence observed as species age. Their study unveiled how alterations in the senescent microenvironment profoundly influence the organism's response to genetic mutations, consequently promoting the positive selection of oncogenic mutations—phenomena repressed in youth but favored in later stages of life (Henry et al., 2010, 2015). Present‐day research is increasingly emphasizing the intricate interplay between aging and carcinogenesis, with particular attention directed toward unraveling the role of systemic or local aging TME in cancer development. In aging individuals, the organismal TME varies, leading to stromal microenvironment reprogramming, ultimately affecting tumor growth and progression (Hessmann et al., 2020).

This review aims to analyze the effects of aging on the TME and its subsequent impact on tumor progression. Special attention will be devoted to investigating the roles of various components within the TME, including stromal cell populations, immune systems, vascular systems, and the extracellular matrix. Furthermore, we will examine how the senescent microenvironment influences the efficacy of treatments such as radiotherapy, immunotherapy, and surgery, aiming to uncover correlations between aging, tumor prognosis, and therapeutic adaptations. Given the global trend towards an aging population, our objective is to utilize this comprehensive analysis to identify additional treatment options for the growing number of elderly cancer patients.

## 2 AGING‐RELATED BIOLOGICAL CHANGES

**FIGURE 2:**
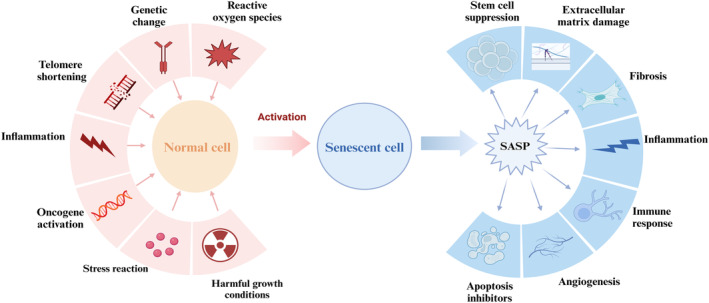
Factors affecting the aging of human cells and their consequences. The factors that promote cellular senescence (pink) are shown in the figure, along with the effects on the organism caused by the senescence‐associated secretory phenotype (SASP) (blue) produced by senescent cells.

Human aging is predominantly attributed to cellular senescence, a state triggered by various factors such as DNA damage, telomere shortening, activation of oncogenes, and cellular injury induced by reactive oxygen species (ROS) (Wyld et al., 2020). The accumulation of senescent cells in tissues, notably in adipose tissue, muscle, and skin, escalates with advancing age (Tchkonia et al., 2010). Adipose tissue is associated with longevity and age‐related metabolic dysfunction (Mau & Yung, 2018). The accrual of aged cells within adipose tissue precipitates a cascade of physiological alterations, elevating the risk of conditions such as diabetes, hypertension, cancer, cognitive impairment, cardiac events, and atherosclerosis (Lutz et al., 2008). As senescent cells accumulate with age, skeletal muscle undergoes a reduction in size and strength, rendering it more susceptible to injuries and impeding its ability to recover from such injuries. This phenomenon may be attributed to the activation of adaptive responses in skeletal muscle triggered by elevated levels of free radical production (Thirupathi et al., 2020). Moreover, exposure to ultraviolet radiation accelerates the aging process of the skin, promoting matrix degradation and diminishing collagen synthesis. These effects can increase the risk of skin cancer (Salminen et al., 2022). Additionally, the extent of human biological aging is influenced not only by genetic factors but also by lifestyle habits (e.g., high‐sugar and high‐fat diets, exercise, and sleep quality) and environmental factors, elucidating the disparity between biological and chronological age for some individuals (Chedraui & Pérez‐López, 2013; Deelen et al., 2019) (Figure 2).

Currently, aging is subject to increasing scrutiny as a heterogeneous cellular state (Ogrodnik et al., 2019). Senescence can be categorized into acute and chronic forms, contingent on kinetics and functionality (Dodig et al., 2019). Acute senescence ensues in response to external stimuli that target specific cell populations within tissues, typically serving to coordinate biological processes like wound healing and tissue repair. This is followed by the recruitment of immune cells to facilitate the clearance of senescent cells, a protective mechanism for the organism (van Deursen, 2014). In contrast, chronic senescence arises due to cellular stress or damage, resulting from the failure of immune cells to promptly remove senescent cells, thereby allowing senescence to progress further (van Deursen, 2014). Chronic aging has been linked to an increased susceptibility to diseases such as atherosclerosis, inflammation, diabetes, and neurodegenerative conditions (Wyld et al., 2020).

**FIGURE 3:**
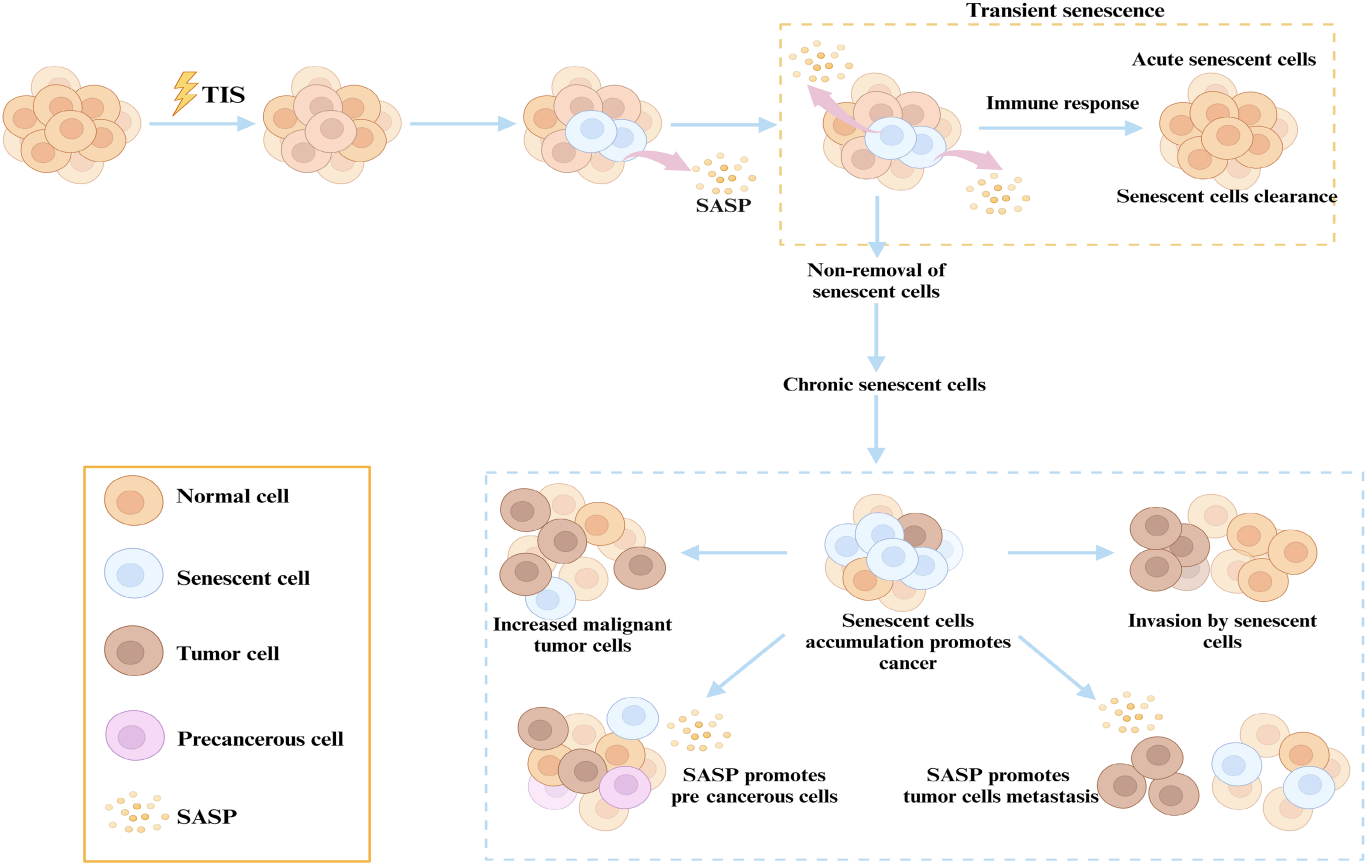
Effects and consequences of therapy‐induced senescence on cellular senescence in the organism. The figure illustrates that therapy‐induced senescence (TIS) can lead to cellular senescence, with some senescent cells transforming into a transient senescent state (acute senescence). The organism then removes these senescent cells by immune surveillance. If these acute senescent cells are not removed, these senescent cells will enter a chronic senescent state, which in turn produces senescence‐associated secretory phenotype (SASP) accumulation, which may lead to the development of precancerous cells. This can further promote the formation, invasion, and metastasis of tumor cells.

Additionally, research has shown that senescent cells induced by oncogene mutations are primarily detected in the early stages of cancer. Over time, tumors must surpass this initial state of senescence to advance into malignant tumors (Saretzki, 2010). This implies that tumor progression is intricately linked to the transition to chronic senescence (van Deursen, 2014). During chronic senescence, senescent cells undergo a variety of changes, including the release of SASP, which can have a bidirectional effect on tumor development (Birch & Gil, 2020; Ou et al., 2022).This role of SASP appears to depend on a variety of factors, such as induction and duration of senescence, specific SASP components, tissue type, and disease background (Rao & Jackson, 2016; Schosserer et al., 2017) (Table 1; Figure 3).

**TABLE 1:** Role of senescence‐associated secretory phenotype (SASP) produced by senescent cells in the tumor microenvironment.

Senescent cell	Aging‐inducing factors	Target of SASP	Major roles of SASP	References
Hepatic stellate cell	High‐fat diets	NK cells M1 macrophages	Impaired antitumor function Senescent cell removal Fibrosis reduction	Krizhanovsky et al. (2008); Loo et al. (2017); Yoshimoto et al. (2013)
Hepatocyte	Oncogene‐induced senescence	CD4+ T cells	Tumor suppression Senescent cell removal	Kang et al. (2011)
Thymic endothelial	Chemotherapy	Lymphoma	Lymphoma recurrence with poor prognosis	Gilbert & Hemann (2010)
Prostate epithelial cells	TIS (docetaxel) PTEN loss	MDSCs	Tumor promotion	Di Mitri et al. (2014); Toso et al. (2014)
Osteoblast	TIS (radiation)	NKT cells	Tumor suppression NKT cell acquisition	Kansara et al. (2013)
Pancreatic duct cells	TIS (MEK and CDK4/6 inhibitors) Ras	M1 macrophages	Tumor suppression Increase drug delivery efficiency	Lesina et al. (2016)
Colon cells	Wnt activation	Colon cells	Durable growth arrest	Pribluda et al. (2013)
Thyroid follicular cells	BRAF	NA	Anoikis resistance NA	Kim et al. (2017)

## 3 SENESCENCE‐RELATED SECRETORY PHENOTYPES

Oncogene‐induced senescence is an effective form of tumor suppression (Acosta et al., 2013). During this process, senescent cells produce a complex inflammatory response known as the SASP (Georgilis et al., 2018).The SASP consists mainly of various inflammatory factors, growth factors, and proteases including interleukins, vascular endothelial growth factor, and matrix metalloproteinases (Gao & Pickett, 2022).The acquisition of the SASP stems mainly from the elevated levels of transcription and protein synthesis within senescent cells. This phenotype leads to non‐autonomous transmission of damage signals from senescent cells to neighboring cells, causing damage to nearby non‐senescent cells and the extracellular matrix. This in turn triggers inflammation and apoptosis of normal cells (Kuilman et al., 2008; Sikora et al., 2021).

SASP production and tumorigenesis are closely related to telomeres. Telomeres, which consist of repetitive DNA and telomere‐binding proteins known as the shelterin complex in human cells, serve as protective caps at chromosome ends, crucial for maintaining genomic stability by preventing erroneous DNA repair (Sui et al., 2020). As organisms age, tumorigenesis becomes intricately linked with telomere dynamics. The process of cell division and senescence often coincides with telomere shortening within the nucleus. Moreover, cells affected by DNA damage response (DDR) are prone to acquiring mutations in genes closely associated with tumorigenesis. Studies have shown that cancer cells activate the telomere maintenance mechanism through two pathways: telomerase‐mediated telomere maintenance (85%) and the alternative lengthening of telomere pathway (15%). These pathways are key in conferring immortality to cancer cells (Gao & Pickett, 2022). Normally, in healthy individuals, a repairable amount of DNA damage does not lead to SASP secretion, which is only initiated by extensive DNA damage (Rao & Jackson, 2016). When tumor cells undergo a severe DNA damage response, senescent cells secrete cytokines and other components of the SASP, recruiting immune cells to remove these damaged or oncogene‐expressing cells, thereby helping to inhibit tumor progression (Kale et al., 2020). However, some cells are capable of promoting tumor progression through the paracrine secretion of pro‐oncogenic cytokines and inhibition of immune cell aggregation (Rao & Jackson, 2016). This suggests that targeting specific cells to secrete SASP to inhibit tumor progression holds great promise.

**FIGURE 4:**
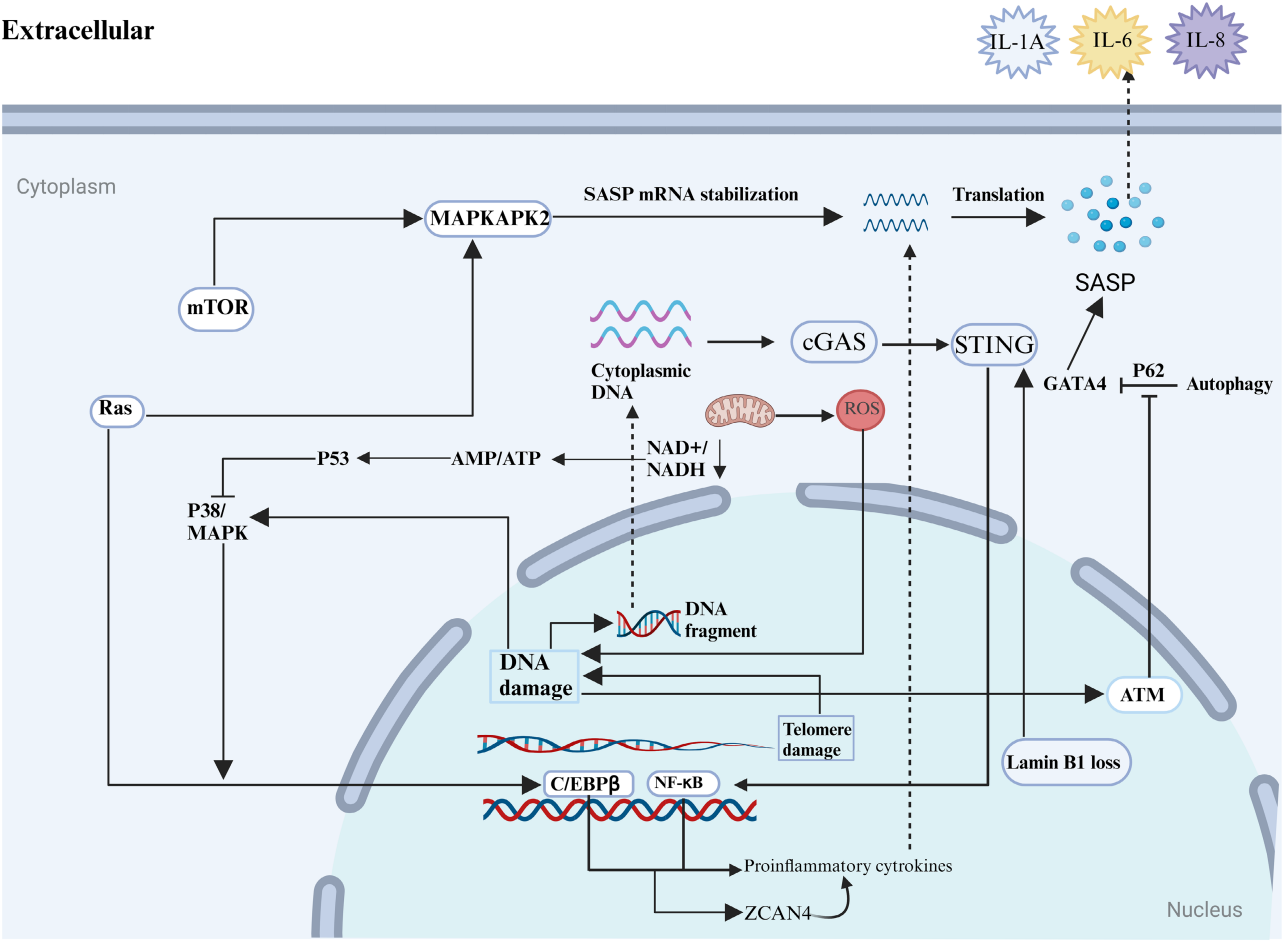
Pathways and mechanisms involved in senescence‐associated secretory phenotype (SASP) production. SASP production in senescent cells. SASP production involves various metabolic pathways and mechanisms, with most pathways culminating in the production of inflammatory cytokines via transcription factors NF‐κB and C/EBPβ. ZCAN4 also induces inflammatory cytokines through NF‐κB. Furthermore, up‐regulation of Ras can regulate SASP production through the translation of mTOR‐regulated MAPKAPK2. The deletion of P53 also leads to SASP production via NF‐κB and C/EBPβ. Reactive oxygen species, by‐products of the mitochondrial electron transport chain in aerobic cells, accumulate during senescence and cause DNA damage, which promotes SASP production. Additionally, mitochondrial damage leads to changes in AMP/ATP and NAD+/NADH ratios, further inducing inflammatory cytokines through NF‐κB and C/EBPβ. DNA fragments from DNA damage and the deletion of lamin B1 promote inflammatory cytokine secretion via the cGAS‐STING pathway. Conversely, cellular autophagy can inhibit SASP production through the P62‐mediated inhibition of the GATA4 gene.

Senescence, similar to many other biological processes, is influenced by multiple signaling pathways and transcription factors. Among them, the NF‐κB and mTOR signaling pathways play a key role, with multiple enhancer regions of SASP factors being transcribed by factors such as NF‐κB (Perluigi et al., 2015; Salminen et al., 2012). SASP is regulated under the control of the mTOR signaling pathway through the translation of MAPKAPK2. Additionally, the deletion of P53 and the upregulation of RAS can also promote the paracrine pathway of SASP (Salminen et al., 2012). ROS, a by‐product of the electron transport chain in aerobic cells, can lead to cellular damage and increased genomic instability when present at high levels, resulting in the activation of NF‐κB, which promotes the production of SASP (Nelson et al., 2018). SASP is essential in promoting the secretion of inflammatory and chemokine factors, angiogenesis, and the growth and degradation of the extracellular matrix (ECM) (Faget et al., 2019) (Figure 4).

## 4 IMPACT OF AGING ON TME

### 4.1 Extracellular matrix

The TME is comprised of the tumor's nonmalignant cells, which include mainly cancer‐associated fibroblasts (CAFs), endothelial cells, pericytes, adipocytes, immune and inflammatory cells, bone marrow‐derived cells, and the ECM. The ECM consists of structural proteins that establish cross talk with the tumor (Belli et al., 2018). It forms a part of the tissue and organ skeleton, composed of supramolecular aggregates of extracellular proteins, proteoglycans, and glycoproteins. These elements exhibit various physical properties like density, stiffness, and tension. These properties can be altered by the cross‐linking and arranging of structural proteins (Eble & Niland, 2019; Karsdal et al., 2013). Additionally, the ECM plays a crucial role in regulating several biological functions, including survival, adhesion, cell proliferation and differentiation, and cell binding (Trapani et al., 2017).

In senescent cells, the ECM undergoes degradation and deformation. It also experiences upregulation or downregulation of its contents. These changes can affect cellular functions, including the normal regeneration of stem cells (Kurtz & Oh, 2012; Levi et al., 2020). In the ECM of senescent tumor stroma, fibroblasts can differentiate into CAFs. These CAFs not only alter the ECM but also promote tumor cell proliferation (Eble & Niland, 2019). This is primarily because the altered ECM can release growth factors and chemokines, like insulin‐like growth factor, fibroblast growth factors, and transforming growth factors. These stimulate adipocytes, Mesenchymal stem cells (MSCs), pericytes, and other cells to further produce CAFs. CAFs are capable of inducing the remodeling of the ECM through the secretion of matrix metalloproteinases, creating an environment conducive to tumor growth (Eble & Niland, 2019; Mao et al., 2021; Sandiford et al., 2018).

Furthermore, cellular senescence often disturbs the synthesis of collagen, fibronectin, elastin, and laminin. Collagen, comprising up to 75% of the ECM, can lead to ECM fibrosis. Aging‐induced reductions in hyaluronic acid (HA) hydration exacerbate this fibrosis, leading to tissue dysfunction (Jo et al., 2021). Fibronectin, an important ECM component, includes the extra domain A, extra domain BHA, and IIICS structural domains. However, factors like hypoxia, mutations, and nutritional deficiencies can result in structural mismatches of fibronectin, particularly of the EDA and EDB types, known as the placenta‐superficial type. This form is expressed at elevated levels in tumor tissues but is hardly present in normal tissues. This suggests that developing a vaccine against EDA and EDB may hold promise for treating and preventing certain cancers (Kumra & Reinhardt, 2016; Zhao et al., 2023).

### 4.2 Angiogenesis

Angiogenesis is crucial for tumor development and essential for the metastatic expansion of solid tumors (Wang et al., 2018). Solid tumors, when exceeding a diameter of 2 mm, rely on the generation of new blood vessels to ensure a continuous blood supply for their survival (Folkman, 1971). The TME is marked by aberrant vasculature, characterized by vessels exhibiting diverse morphologies, high perfusion efficiency, and increased permeability (Vimalraj, 2022).

In conventional tumor angiogenesis, three primary types are recognized, with sprouting angiogenesis involving the differentiation of endothelial progenitor cells into endothelial cells, ultimately forming a neovascular network (Ronca et al., 2017). Another mechanism involves the proliferation of existing vascular endothelial cells to create new blood vessels (Naito et al., 2020). Recent research has unveiled the significance of angiogenic mimicry (VM) in providing an endothelium‐independent blood supply to tumor cells, crucial for tumor progression (Luo et al., 2020). VM strongly associates with the malignancy grade and prognosis of tumors, serving as the primary blood supply in early‐stage tumors, later replaced by alternative angiogenic processes (Chen & Chen, 2014; Luo et al., 2020). The limited efficacy of some clinical treatments targeting tumor angiogenesis in recent years may be attributed to VM activation.

To identify commonly shared markers of senescent cells, researchers established eight different senescence models using various triggers on in vitro cell lines, including human diploid fibroblasts (WI‐38, IMR‐90) and endothelial cells (HUVEC, HAEC). They observed upregulation of 251 transcripts, predominantly encoding proteins such as SRPX, SRPX2, and SRPX1 mRNAs (Casella et al., 2019). Several of these transcripts have dual associations with both aging and cancer. For instance, SRPX mRNA expression is heightened in senescent cells but diminished in tumor cells (Shimakage et al., 2009; Tambe et al., 2004). Consequently, promoting SRPX mRNA expression is closely linked to inhibiting tumor progression and promoting senescence. SRPX2 exhibits the potential to stimulate vascular regeneration through the uPAR and integrin/FAK pathways, suggesting its involvement in tumor vascular regeneration (Tambe et al., 2007). Ruscetti et al. (2020) identified that senescent cells acquire SASPs, which encompass numerous pro‐angiogenic and pro‐inflammatory factors, including vascular endothelial growth factor, platelet‐derived growth factor, and CC chemokine ligand 5, contributing to vascularization and remodeling during induced senescence in a mouse model of pancreatic ductal adenocarcinoma. Furthermore, SASP mediates the activation of vascular endothelial cells, thereby stimulating CD8+ T cells and enhancing tumor sensitivity to the PD‐1 checkpoint (Ruscetti et al., 2020). These intricate interactions underscore the multifaceted nature of the TME in aging and tumor progression.

### 4.3 Other tumor microenvironment components

Current research into aging has predominantly concentrated on fibroblast‐associated tumor pathology because fibroblasts constitute the most prevalent component of the aging TME. Nevertheless, it is imperative to acknowledge the presence of various other senescent cell populations within the aging TME, encompassing endothelial cells, epithelial cells, immune cells, stem cells, and specific tumor cells (Fane & Weeraratna, 2020). These diverse cell populations secrete substantial quantities of SASP factors, which exert direct regulatory effects on neighboring cells, while also contributing to inflammation and tumor development, albeit concurrently inducing senescence (Faget et al., 2019; Franceschi & Campisi, 2014). An intriguing study by Baker et al. (2011) introduced a novel transgenic model named INK‐ATTAC, designed to target the senescence marker p16Ink4a. Remarkably, administration of this model led to a profound reduction in p16Ink4a‐expressing senescent cells, consequently resulting in a significant decrease in tumor formation. This intriguing outcome challenges the prevailing notion that bypassing or evading senescence is an imperative step in tumorigenesis, suggesting that the elimination of senescent cell populations can curtail the initial tumor cell pool (Moiseeva et al., 2022).

Several instances in current research emphasize the bidirectional influence of senescent cell populations on tumor development, contingent upon the specific context (Vernot, 2020). For instance, Liedtke et al. (2015) investigated triple‐negative breast cancer and discovered that tumors in older mice (aged 10 months or more) exhibited a comparatively slower growth rate and reduced metastatic potential in contrast to younger mice (8–10 weeks old). Further investigations unveiled that this phenomenon primarily stemmed from the upregulation of calnexin‐independence factor 1 receptors and the secretion of growth factor granule proteins in young mice, which substantially promoted robust tumor growth and metastasis. Intriguingly, the transplantation of bone marrow‐derived cells from young mice into older mice activated the TME and consequently facilitated tumor progression (Marsh et al., 2016). These paradoxical findings underscore the contextual nature of the effects exerted by various senescent cell populations within the aging TME on tumor progression.

## 5 APPLICATION OF AGING IN CLINICAL TUMOR THERAPY

### 5.1 Chemotherapy

Chemotherapy, as a nonspecific and aggressive therapeutic approach, primarily targets the malignant proliferation of cells, often leading to apoptosis and subsequent tumor regression (Boohaker et al., 2012). This effect is largely attributed to the capacity of chemotherapy to induce DNA damage, including DNA strand breakage or cross‐linking, which triggers cell death through the DDR mechanism (van Deursen, 2014). Furthermore, chemotherapy can also initiate the senescence process in cells through nonlethal DDR activation. Interestingly, moderate doses of chemotherapy are more prone to induce cellular senescence compared to higher doses (Wyld et al., 2020). Chemotherapy‐induced senescence in cancer treatment is a multifaceted phenomenon encompassing both acute and chronic senescence. Acute senescence results from persistent DDR activation in cells, while chronic senescence primarily arises from mild genotoxic stress. Over time, these senescent cells transition from a state of mild stress to a state of prolonged cell cycle arrest (Le et al., 2010; Roninson, 2003).

**FIGURE 5:**
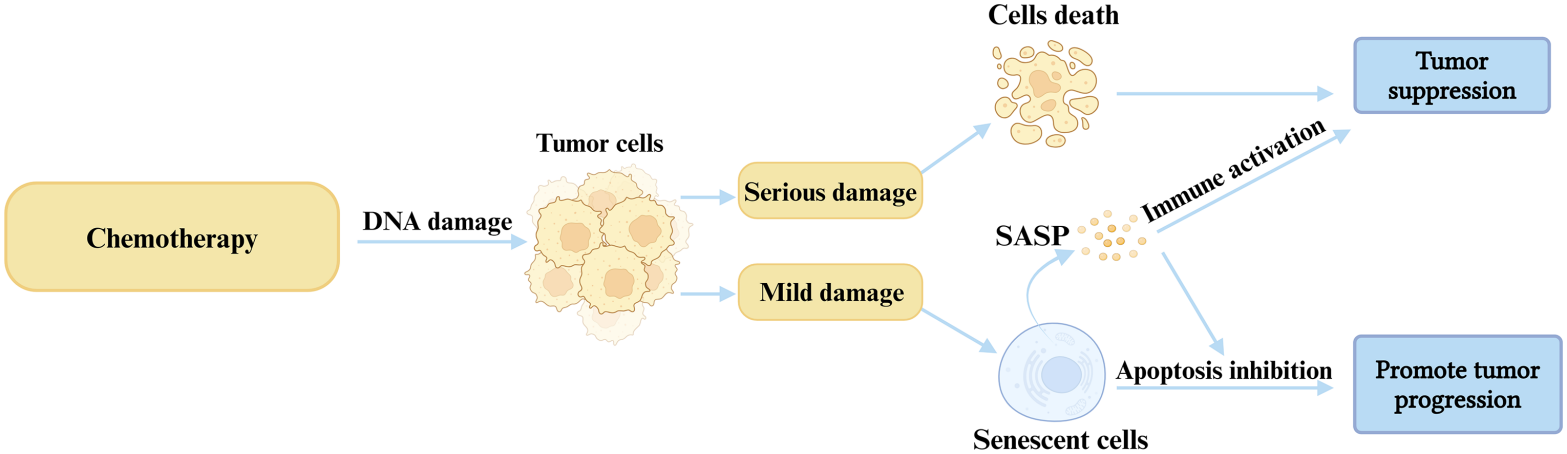
Effects of chemotherapy on tumor cell senescence and outcomes. Chemotherapy functions by damaging the DNA of malignant tumor cells. Cells that incur severe damage enter a death cycle, while the surviving tumor cells enter a senescence cycle and produce senescence‐associated secretory phenotype (SASP). This contributes to tumor suppression by activating the immune response and inhibiting apoptosis.

Nonetheless, chemotherapy often elicits various side effects, some of which pose a threat to patients' lives. For instance, if chemotherapy fails to induce the death of all target cells but instead triggers senescence in some of them, the SASP released by these senescent cells may contribute to tumor recurrence (Basisty et al., 2020). Notably, Demaria et al. (2017) have shown that therapy‐induced senescence (TIS) induced by various chemotherapeutic agents in senescent fibroblasts leads to a cascade of inflammatory responses. Eliminating these senescent fibroblasts significantly reduces chemotherapy‐induced side effects and diminishes the likelihood of cancer recurrence. Moreover, chemotherapy can give rise to off‐target effects, including damage to stem cells and bone marrow function (Dobrenis et al., 2015). Studies have revealed that treating mice of different ages with Adriamycin demonstrated varying resistance to chemotherapeutic drug toxicity in MSCs, with middle‐aged mice displaying greater resistance and older mice experiencing more pronounced side effects (Bashiri Dezfouli et al., 2017). This suggests the possibility of mitigating off‐target effects by aligning patient age with stem cell transplantation in conjunction with chemotherapy treatment, although careful consideration of safety thresholds is warranted, particularly in the elderly population (Bashiri Dezfouli et al., 2017). Furthermore, high‐dose chemotherapy and stem cell transplantation can influence the expression of senescence markers, as evidenced by a significant increase in p16INK4a expression in T cells observed 6 months posttransplantation, with greater effects in autologous as opposed to allogeneic patients (Wood et al., 2016). These findings suggest a potential association between this therapy and T‐cell aging. Additionally, the thymus damage caused by high‐dose chemotherapy before transplantation leads to impaired thymic function and accelerated thymic senescence (Min et al., 2005; Montecino‐Rodriguez et al., 2013). Taken together, these insights point toward the prospect of targeting chemotherapy‐induced aging as a means to reduce side effects and offer novel avenues for future treatments (Figure 5).

### 5.2 Radiotherapy

Radiation therapy (RT) stands as a cornerstone among contemporary cancer treatment modalities, with over 70% of tumor patients undergoing RT intervention (Zhou et al., 2023). The fundamental mechanism underlying RT's efficacy primarily revolves around direct DNA damage and its impact on tumor cells, mediated by the generation of ROS (Zou et al., 2017). Following exposure to RT, surviving cells typically manifest in two distinct outcomes: they either acquire resistance to radiation and persist in their regenerative capacity, or they succumb to severe damage and perish (Tsolou et al., 2019). Regenerated cancer cells often display “stem cell” characteristics, potentially fostering a milieu conducive to chronic and aggressive recurrence (Milanovic et al., 2018).

In the context of glioblastoma, research has revealed a fascinating phenomenon. Glioblastoma cells that withstand lethal doses of radiation, due to innate resistance, tend to undergo homotypic cell‐to‐cell fusion. This results in a multinucleated and giant cell phenotype (Kaur et al., 2015). Furthermore, this process induces multinucleated and giant cell phenotype cells to enter a state of senescence and subsequently release SASP factors. This mechanism is postulated to contribute significantly to glioblastoma relapse (Kaur et al., 2015).

Radiation therapy also provokes an immune response. During the progression of tumors, malignant cells often undergo immune editing, which diminishes their antigenicity and adjuvant properties. Interestingly, RT has the potential to enhance this immune‐editing process, possibly through the production of SASP by senescent cells induced by RT (Wennerberg et al., 2017). Moreover, exposing mice to sublethal doses of infrared light for up to 45 weeks increased DNA damage foci and elevated p16 (INK4a), potentially contributing to declining bodily functions in tumor‐surviving patients (Le et al., 2010). Thus, further research is imperative to unravel the intricate interplay between RT, the immune system, and senescence, shedding light on the intrinsic connections among these components.

### 5.3 Surgeon therapy

Surgical intervention remains crucial in treating solid tumors, with a near‐universal application across various treatment strategies (Chen et al., 2019). Postsurgical wound healing, however, is complex, influenced by both positive and negative aspects of aging (Guo & Dipietro, 2010). Aging is a critical determinant in wound healing, often prolonging the process without adversely affecting the overall quality of repair (Gosain & DiPietro, 2004). This delay, particularly notable in the elderly following surgery, is attributed to altered inflammatory responses, crucial for wound healing phases such as hemostasis, cellular migration, differentiation, and subsequent processes like collagen synthesis and tissue remodeling (Wyld et al., 2020).

A key observation in elderly individuals is the delayed T‐cell infiltration in primary wounds, a factor that contributes to slower wound healing. This phenomenon leads to diminished chemokine production and macrophage phagocytosis (Swift et al., 2001). Demaria et al. (2014) have identified that senescent fibroblasts and endothelial cells initially appear in skin wounds, subsequently expediting wound healing by inducing myofibroblast differentiation through the secretion of growth factor AA, a component of the SASP.

Senescence plays a dual role, facilitating wound healing and potentially halting it to avert excessive fibrosis. In the final wound healing stages, the matricellular protein CCN1 is pivotal, inducing senescence in fibroblasts and the expression of antifibrotic genes (Jun & Lau, 2010). The exacerbation of fibrosis in CCN1‐deficient mice underscores its indispensable role in wound repair. Hence, understanding and managing wound healing in the aging population, particularly post‐resection surgery, is crucial, given the growing number of elderly individuals and their unique medical needs.

### 5.4 Immunotherapy

Cancer immunotherapy, a field that has seen rapid evolution in recent decades, represents a paradigm shift in oncology, enabling targeted modulation of the immune microenvironment to eradicate tumor cells (Tang et al., 2021). Despite its transformative potential, a notable subset of patients remains unresponsive, often due to the emergence of primary and acquired resistance mechanisms (Sharma et al., 2017). With age‐related changes in the immune system, tailoring immunotherapeutic approaches to the immunological profiles of the elderly is crucial.

Current clinical applications and trials frequently utilize immune checkpoint inhibitors, targeting proteins such as programmed cell death 1, programmed death‐ligand 1 (PDL1), and cytotoxic T‐lymphocyte‐associated antigen 4 (Zhang et al., 2021). These targets are integral in the mechanism by which cancer cells evade immune detection, essentially inducing a dormant state in immune cells during cancer proliferation (Qin et al., 2019; Wieder et al., 2018). Notably, age‐associated increases in PD1 expression, particularly in T cells, have been documented, suggesting a unique vulnerability in the elderly (Shimada et al., 2009). This finding underscores the potential of PD1 inhibitors in rejuvenating T‐cell functionality in this demographic. The mTOR inhibitor rapamycin, for instance, has shown promise in reducing age‐related PD1 elevation, boosting antigen‐specific immunity, and mitigating immune system aging (Hurez et al., 2015). Thus, integrating anti‐PD1 therapies with strategies to inhibit B7‐H1 (also known as PDL1) expression is critical in customizing immunotherapy for elderly cancer patients (Herbst et al., 2014).

Aging also correlates with significant changes in immunomodulatory factors such as indoleamine 2,3‐dioxygenase 1 and PD‐L1, particularly within the brain and dendritic cells. This period is marked by an upsurge in suppressor regulatory T cells and decrease in CD8+ T cells (Ladomersky et al., 2019). Such trends suggest a link between aging and increased immunosuppression in the nervous system, potentially impacting the efficacy of immunotherapy in elderly patients (Ladomersky et al., 2019, 2020).

In addition, age‐associated TME can dampen the antitumor immune response and facilitate tumor cell evasion from immune surveillance (Xia et al., 2023). This phenomenon also impacts the efficacy of immunotherapy. Studies have revealed that anticancer treatments trigger the release of significant amounts of ATP, which undergoes dephosphorylation by exonucleases (CD39 and CD73). This process generates elevated levels of immunosuppressive metabolites, such as adenosine, within the TME, thereby aiding tumor cell escape (Faas et al., 2017; Leone & Emens, 2018). Consequently, there is a crucial need to limit ATP release into the extracellular space and to inhibit its degradation to effectively reshape immune responses in cancer immunotherapy (Boison & Yegutkin, 2019). Addressing these age‐related changes, alongside a deeper understanding of the TME and its evolution with age, is essential for improving predictions of patient responses to immunotherapies and identifying more effective biomarkers.

## 6 AGING AND ADAPTABILITY TO TUMOR THERAPY

**FIGURE 6:**
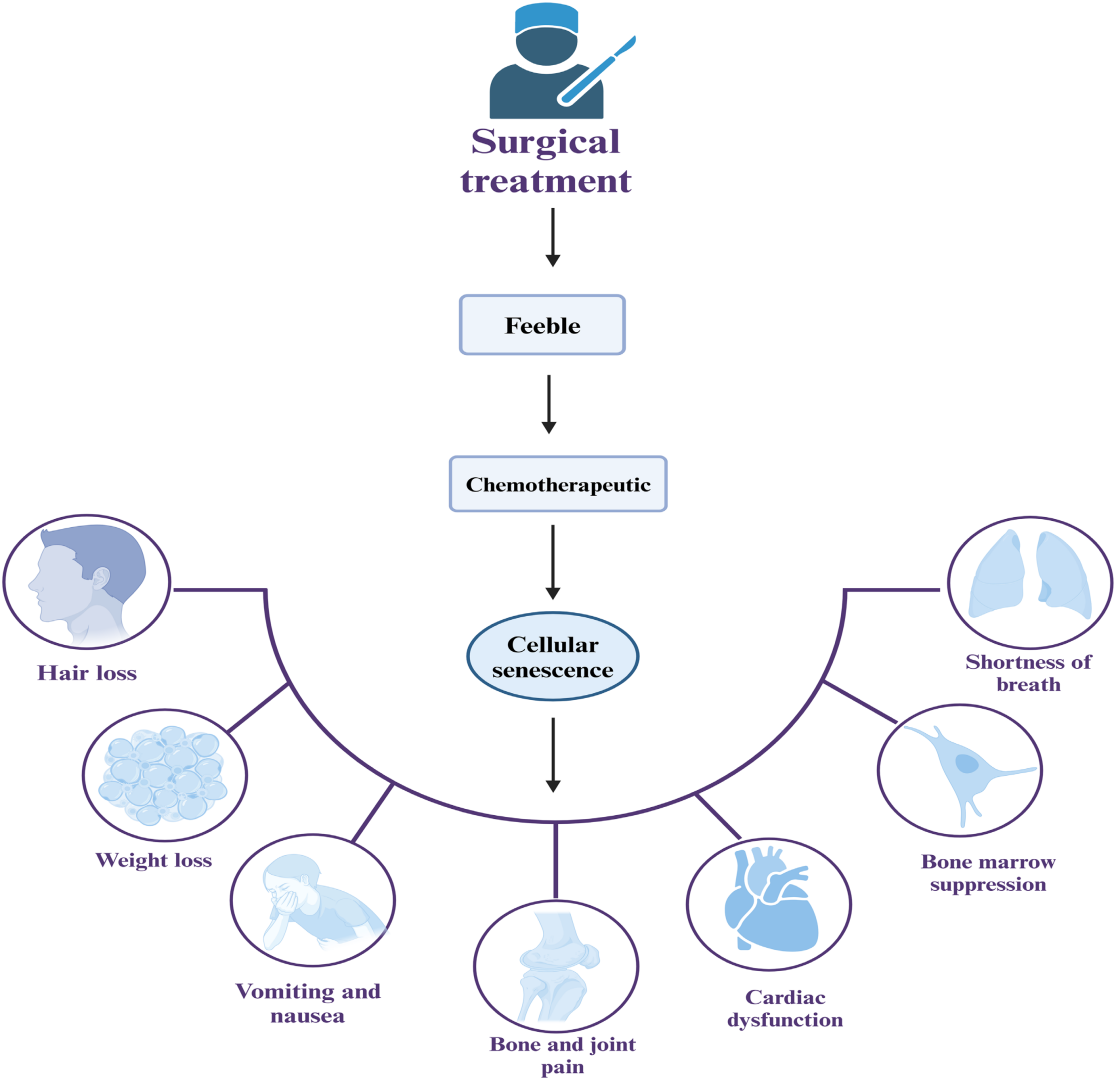
Prognosis of aging and tumor therapy. Elderly oncology patients who have undergone tumor removal surgery often experience physical debility. This debility can impact the body's ability to adapt to chemotherapy and increase the incidence of chemotherapy complications.

Elderly cancer patients frequently pose intricate therapeutic challenges due to the concurrence of co‐morbidities, age‐related organ impairments, and frailty (Colloca et al., 2020). Notably, frailty, observed in an estimated 25% of individuals undergoing surgical oncology, is associated with extended hospital stays and protracted postoperative recuperation (Bratzke et al., 2018; Moug et al., 2016). This susceptibility is further accentuated in chemotherapy contexts, where frailty may exacerbate the risk of treatment‐related complications (Adjogatse et al., 2014) (Figure 6).

In a pivotal investigation, Xu et al. (2018) demonstrated the systemic consequences of cellular senescence. Their study involved introducing senescent cells into youthful mice, leading to pronounced physical debilitation and a significant reduction in lifespan. In contrast, treatment with a senolytic combination of dasatinib and quercetin resulted in substantial diminution of senescent cell populations, functional amelioration, and a marked prolongation of survival. This was evident not only in young mice engrafted with senescent cells but also in naturally aging mice. These results highlight the deleterious impact of senescent cells and the therapeutic potential of targeting these cells for removal. In addition, chemotherapeutic agents induce senescence in human progenitor cells, and the resulting SASPs can cause systemic or localized inflammation. Eliminating these senescent cells significantly reduces the side effects of the drugs on the body, including bone marrow suppression, cardiac dysfunction, cancer recurrence, and frailty (Demaria et al., 2017). Collectively, these insights suggest that addressing senescence‐induced dysfunctions is crucial for bolstering physiological resilience in post‐cancer treatment scenarios.

## 7 CORRELATION BETWEEN AGING AND TUMOR PROGNOSIS

The prevalence of senescent cells in normal tissues such as skin and adipose tissue notably increases with age in older adults (Jeyapalan et al., 2007; Krishnamurthy et al., 2004; Waaijer et al., 2012). These cells are not only abundant in normal aging tissues but are also present in cancers and precancerous lesions, where they have been investigated as potential prognostic markers. The expression profiles of these cells escalate progressively from normal to benign, and further to precancerous states (Pare et al., 2019). Senescence markers have been validated in a variety of tumors. Key senescence markers include β‐galactosidase, p16INK4A, p21, and heterochromatin levels often assessed alongside markers of proliferation inhibition (González‐Gualda et al., 2021; Gorgoulis et al., 2019). The modulation of these markers, either upregulation or downregulation, is observed during the oncogenic process, suggesting their integral role in cancer development and progression. In breast cancer, for instance, p16 levels have been correlated with cancer subtype, prognosis, and clinicopathologic factors indicating a significant role in disease trajectory (Shin et al., 2015). Furthermore, the expression of p16INK4a and p14ARF in breast cancer has been associated with cancer transformation and poorer prognostic outcomes (Pare et al., 2016). Contrarily, a study by Althubiti et al. (2014) utilizing an online tool for the aggregated analysis of the GEO database revealed a strong association between high expression of senescence markers and favorable prognosis across various cancers, including colorectal, lung, glioma, breast cancer, and lymphoma. This relationship was particularly pronounced in breast cancer, with liver and kidney cancers also showing significant correlations. Notably, decreased levels of these markers were linked to poor prognosis (Macher‐Goeppinger et al., 2013; Xiang et al., 2019). These findings collectively underscore the importance of senescence markers in cancer prognosis, highlighting their potential utility as prognostic tools across multiple cancer types (Table 2).

**TABLE 2:** Relationship between aging markers and tumor prognosis.

Cancer type	Prognosis	References
Breast cancer	Prognosis varies with different aging markers	Althubiti et al. (2014); Pare et al. (2016)
Hepatocellular carcinoma	Aging is associated with a poorer prognosis	Macher‐Goeppinger et al. (2013); Xiang et al. (2019)
Renal cancer	Aging is associated with a poorer prognosis	Macher‐Goeppinger et al. (2013)
Colorectal cancer	Aging is associated with a better prognosis	Althubiti et al. (2014); Roxburgh et al. (2013)
Hodgkin lymphoma	High expression levels for senescence markers are associated with better prognosis	Caliò et al. (2015)
Glioma	High expression of senescence markers associated with improved prognosis	Althubiti et al. (2014)
Lung cancer	High expression of senescence markers associated with improved prognosis	Althubiti et al. (2014)

Recent studies have increasingly focused on dormant tumor cells, which share common characteristics with proliferation‐arrested cells such as quiescent and senescent cells (Kirkland, 2023). The term “dormant cancer cells” refers to cancer cells that are able to escape destruction by the immune system and, under the right circumstances, are able to reawaken and proliferate to repopulate the tumor (Truskowski et al., 2023). Over the past two decades, research has explored how senescence serves as a pathway to tumor dormancy (quiescence), ultimately contributing to metastatic recurrence. During treatment, tumor cells surviving therapy enter a senescent dormant state, resisting treatment and becoming highly susceptible to drug resistance, invasiveness, and metastasis, thus laying the groundwork for recurrence (Risson et al., 2020). Disseminated tumor cells (DTCs) originating from early primary lesions often experience a period of dormancy, governed by two key processes. Initially, a single DTC or a small cluster may enter a state of proliferative arrest upon reaching a secondary site. Subsequently, these DTCs might resume proliferation, forming a tumor mass. However, constrained by limited blood and nutrient supply, coupled with an absence of immune surveillance, these DTCs remain below the threshold of clinical detectability (Aguirre‐Ghiso, 2007; Phan & Croucher, 2020). This phenomenon also elucidates the extended latency period preceding tumor recurrence. Moreover, signaling from senescent tumor cells within the metastatic niche triggers a transition from dormancy to heightened aggressiveness and metastatic potential. Hence, the influence of senescence on dormant cancer cells significantly impacts tumor prognosis.

## 8 DISCUSSION AND CONCLUSION

Based on the aforementioned information, aging is a multifaceted phenomenon intricately linked to the cell cycle and the emergence of numerous human ailments, particularly tumors. Traditionally, cellular senescence marks the irreversible halt of division and entry into a state of permanent growth arrest as individuals age, which has been perceived as a mechanism for suppressing tumorigenesis; however, this perspective fails to elucidate the elevated prevalence of cancer among older individuals. The impact of the senescent microenvironment on tumor progression underscores the imperative for a more comprehensive exploration of age's role in tumor therapy.

Age‐related TME possess the capacity to exert a substantial influence on the advancement of tumors, propelling tumor cells from a state of sluggish growth to one characterized by heightened invasiveness and metastasis. These transformations encompass the secretion of diverse factors, alterations in the TME's structure, and even macroscopic changes. Senescence is a key effector mechanism of several chemotherapeutic agents, induced directly on the one hand and through the immunostimulatory effect of SASP on the other hand. However, challenges persist in the study of the aging TME, including the absence of drugs capable of effectively inducing senescence in a substantial portion of cancer cells, efficient discrimination between normal and cancerous cells, and the lack of clear markers distinguishing senescence from growth arrest.

A comprehensive roadmap of strategies and key initiatives is essential to confront the challenges posed by the senescent microenvironment in tumorigenesis. This roadmap will encompass various approaches, including the identification and development of novel biomarkers for early detection of senescent cells within the TME. Developing appropriate criteria for scoring SASP factors is crucial for detecting dormant cancer cells and assessing the burden of senescent cells, enabling the detection of dormant cancer cells following TIS and guiding the use of drugs targeting senescent cells. Furthermore, advancing technologies for real‐time monitoring of tumor‐senescent cell interactions is critical. Interdisciplinary collaborations bridging oncology, gerontology, and immunology are necessary to foster innovative research methodologies and enhance our understanding of the senescent TME. In the future, emerging therapies and drugs targeting the senescent TME are anticipated to enter clinical practice, effectively mitigating the escalating global public health burden by reducing the burden of dormant tumor cells after TIS and preventing recurrence and metastasis.

## AUTHOR CONTRIBUTIONS

WZ and KZ: Conceptualization, Methodology, Data curation and Writing‐original draft preparation. FH and XS: Conceptualization, Supervision, Writing‐reviewing and Editing. JS, HQ, YM and CK: Data Curation and Investigation. All authors agree to be accountable for all aspects of the work.

## CONFLICT OF INTEREST STATEMENT

The authors have no conflict of interest to declare.

## ETHICS STATEMENT

Not applicable.

## Data Availability

Not applicable for review article.
